# Environmental influence on calcification of the bivalve *Chamelea gallina* along a latitudinal gradient in the Adriatic Sea

**DOI:** 10.1038/s41598-019-47538-1

**Published:** 2019-08-01

**Authors:** Arianna Mancuso, Marco Stagioni, Fiorella Prada, Daniele Scarponi, Corrado Piccinetti, Stefano Goffredo

**Affiliations:** 1 Marine Science Group, Department of Biological, Geological and Environmental Sciences, University of Bologna, Via Selmi 3, I-40126 Bologna, European Union, Italy; 2 Laboratory of Fisheries and Marine Biology at Fano, Department of Biological, Geological and Environmental Sciences, University of Bologna, Viale Adriatico 1/N, I-61032, Fano, PU European Union, Italy; 3 Department of Biological, Geological and Environmental Sciences, University of Bologna, Via Selmi 3, I-40126 Bologna, European Union, Italy

**Keywords:** Macroecology, Zoology

## Abstract

Environmental factors are encoded in shells of marine bivalves in the form of geochemical properties, shell microstructure and shell growth rate. Few studies have investigated how shell growth is affected by habitat conditions in natural populations of the commercial clam *Chamelea gallina*. Here, skeletal parameters (micro-density and apparent porosity) and growth parameters (bulk density, linear extension and net calcification rates) were investigated in relation to shell sizes and environmental parameters along a latitudinal gradient in the Adriatic Sea (400 km). Net calcification rates increased with increasing solar radiation, sea surface temperature and salinity and decreasing Chlorophyll concentration in immature and mature shells. In immature shells, which are generally more porous than mature shells, enhanced calcification was due to an increase in bulk density, while in mature shells was due to an increase in linear extension rates. The presence of the Po river in the Northern Adriatic Sea was likely the main driver of the fluctuations observed in environmental parameters, especially salinity and Chlorophyll concentration, and seemed to negatively affect the growth of *C*. *gallina*.

## Introduction

Valuable ecological interactions between organisms and their habitat can be unravelled through studies carried out along latitudinal gradients, where varying environmental pressures can be explored on both biological and evolutionary processes^1^. Latitude implies variations in thermal conditions. Determining the influence of temperature on growth is important given the predicted global climate change scenarios that will likely be thermally challenge for most ectotherms^2^.

Shells of marine molluscs register environmental factors in the form of geochemical properties, shell microstructure and shell growth rates^3^ and, depending on the species and habitat, environmental variables may even be more relevant than physiological ones for shell growth^3,4^.

Previous studies identified several drivers that influence the shell growth rate of bivalves including temperature^5,6^, food supply and quality^7,8^, salinity^9^, latitude^1^ and also reproduction^10^. Evidence for modification in growth in marine molluscs with the environment comes for example from the great scallop *Pecten maximus* along the Northeast Atlantic coast^1^ and from the *Mytilus edulis* along the British coast in the Irish Sea^11^, showing a decrease in shell growth with higher temperatures.

Shell growth occurs as the result of the umbonal-ventral linear extension of the shell per unit time and net calcification rate is the product of shell bulk density (shell mass/volume ratio, including the volume of pores) and linear extension rate. Shell linear extension rates in bivalves decrease through ontogeny^12^ and it might be influenced by seasonality or changes in environmental parameters^13^. Since decreasing linear extension rate is usually accompanied by an increasing in shell bulk density, total CaCO_3_ variations deposited by the organism and linear extension rate may not correlate^13^. Therefore, differences in shell net calcification can result either from constant bulk density and non-continuous linear extension over the year, or modifying bulk density and homogeneous linear extension rate.

Linear extension rates also indicate the required time to reach a determined marketable size^14^. Highlighting the size-age relation is crucial to enforce correct management strategies^15^. The calcareous shell of many bivalves encloses an ontogenetic record in the form of annually resolved growth increments^16,17^ and reliable and accurate information can be revealed on bivalves’ life history such as their age^17,18^.

Bivalve age can be estimated using different methods: mark and recapture experiments^17^, analysis of size-frequency distributions^19^, counting of annual growth marks or rings visible on the shell surface or in the microstructure of shell sections^17,20,21^ and analysis of oxygen isotopic composition along the shell growth direction^15,22^. Visible rings and banding patterns are often formed on the shells of bivalves when they undergo periods of reduced shell growth^23^. Ring forming has been associated with conditions that likely affect growth, such as shifts in habitat parameters (e.g., temperature) or intrinsic conditions (e.g., spawning)^24^.

**Figure 1:**
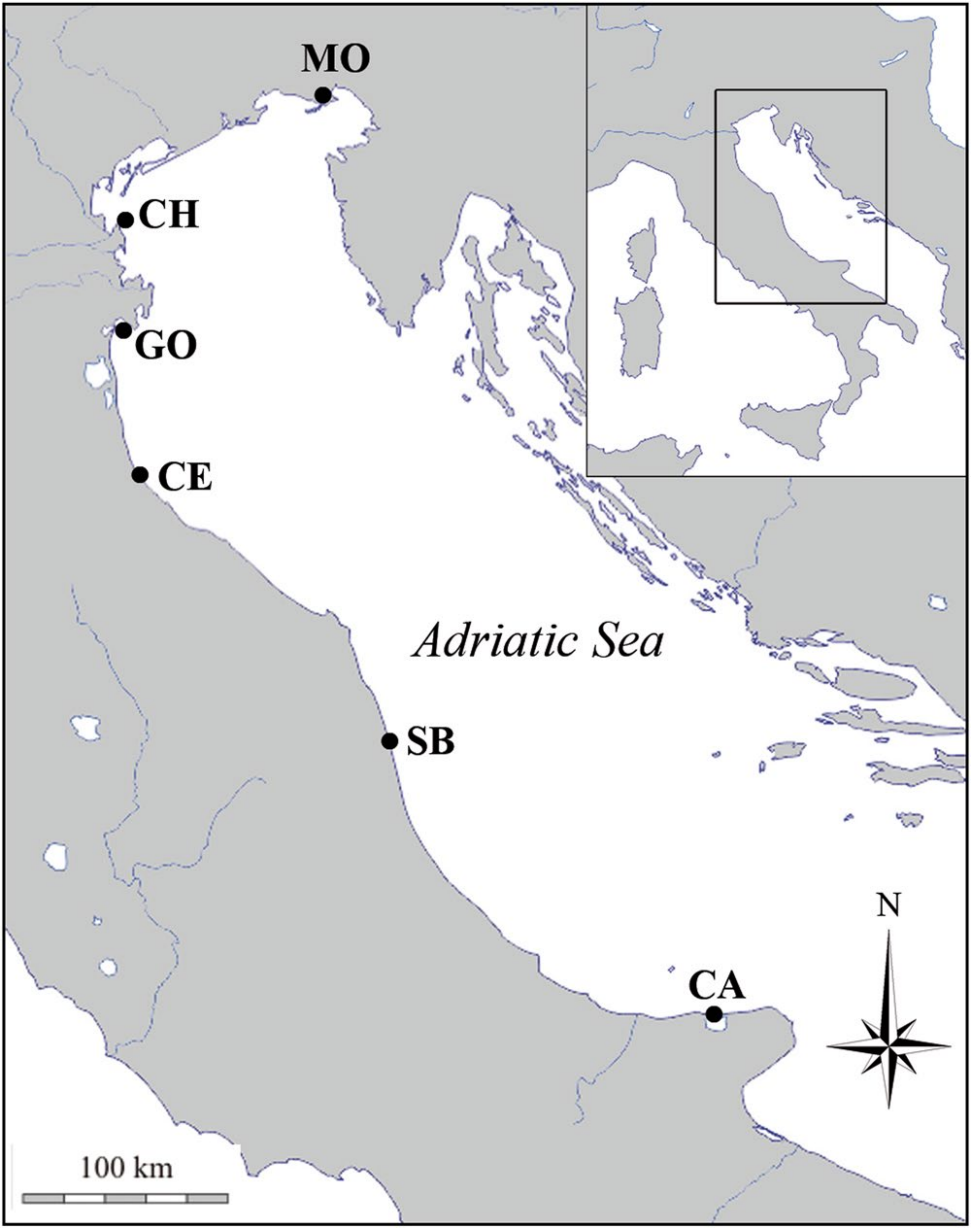
Map of the Adriatic Sea pinpointing the sampling sites of *C*. *gallina*. Abbreviations and coordinates of the sites in order of decreasing latitude: MO, Monfalcone 45°42′N, 13°14′E; CH, Chioggia 45°12′N, 12°19′E; GO, Goro 44°47′N, 12°25′E; CE, Cesenatico 44°11′N, 12°26′E; SB, San Benedetto 43°5′N, 13°51′E; CA, Capoiale 41°55′N, 15°39′E. The map was downloaded from d-maps.com site (http://www.d-maps.com) and modified with Adobe Photoshop CS4.

The present study investigated the shell growth of *Chamelea gallina* in six sites along a latitudinal environmental gradient in the Adriatic Sea (eastern coast of Italy; Fig. 1). *C*. *gallina* (Linnaeus, 1758) is a clam species living mainly in the infralittoral zones of the Black Sea and Mediterranean Sea^25^. Along the Adriatic coast of Italy, *C*. *gallina* is a valuable resource, with a high market price (8–10 € per kg), supporting a relevant commercial dredge fishery^17^. Despite the economic importance of this clam, few studies have investigated the growth of *C*. *gallina* during its lifespan and in relation with environmental variations. A previous study investigated the effects of environmental parameters on shell features of commercial size (>25 mm) at macro, micro and nanoscale levels, at the same six sites and showed that shells of the warmer and more irradiated populations were more porous and less resistant to breakage^26^. Other studies found that temperatures below 10 °C and above 27 °C slowed or inhibited shell linear extension rates of *C. gallina*^19,27,28^.

**Figure 2:**
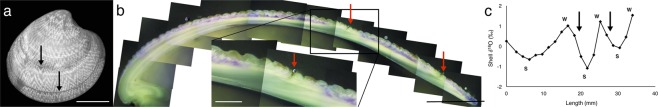
Shell ageing methods. Age determination in the same shell by means of the three methods (**a**). External growth rings (black arrow) on the surface of *C*. *gallina* after shell scanning. Scale bar = 1 cm. (**b**) Internal annual growth bands (red arrows) in the shell section of *C*. *gallina* from the umbo to the ventral margin after Mutvei’s solution treatment. Annual growth lines stood out as etch-resistant ridges, loosely associated with staining. Scale bar = 0.5 cm. Scale bar of the zoom box = 0.1 cm. (**c**) First and second year (black arrows) according to the sequence of lighter (summer, S) and heavier (winter, W) values of δ^18^O along the shell growth axis. This clam likely borned in spring.

Previous studies on the age and shell linear extension rate of *C*. *gallina* in the Mediterranean have employed length-frequency distributions^29^, surface shell rings^30^, shell thin sections^31^ and acetate peels^19,32^. In this study, three different independent ageing methods were used: shell surface growth rings, shell internal bands (shell cross-sections and Mutvei’s solution) and stable isotope composition (Fig. 2). The counting of external rings seems to be fairly accurate method in young shells^19^, as *C*. *gallina* is, reaching 4 years in the Mediterranean Sea, while an accurate age determination in older and thicker shells seems to be difficult^33^. Counting of internal bands in shell sections using Mutvei’s solution is a new and easy-to-use technique for resolving annual growth structures in skeletons of many organisms^34^. Shell oxygen isotope (δ^18^O) along the growth direction provides another clear method of ontogenetic age determination, in which age is directly indicated by the sequence of lower (summer) and higher (winter) δ^18^O values recorded by the shells^15^.

Age and growth studies are the very formal and fundamental components of fisheries management. Although age and growth data are generally considered together, each component provides exclusive and beneficial information on individuals and population. Age estimation offers a favourable knowledge about the features of individual organism in addition to information on the age structure of the entire population. Age and growth studies of commercial marine species are used to study longevity, mortality, productivity, yield and population dynamics that, in turn, are crucial for responsible fisheries management.

The aims of this study were to: 1) determine the growth functions for each site using three independent ageing methods; 2) measure shell skeletal parameters, such as micro-density (mass per unit volume of the shell material, excluding the volume of pores) and apparent porosity (percentage of the pore volume connected to the external surface), together with shell growth parameters, such as bulk density, linear extension and net calcification rates at each site comparing these parameters along a latitudinal gradient of environmental variables in the Adriatic Sea. While the previous study, along the same latitudinal gradient, considered only solar radiation (SR), sea surface temperature (SST) and shells of commercial size (>25 mm)^26^, here we investigated also sea surface salinity (SSS), Chlorophyll concentration (Chl) and shells of all sizes, from immature to mature clams.

## Results

SR, SST and SSS and Chl varied among sites along the latitudinal gradient (Kruskal-Wallis test, df = 5, p < 0.001; Table 1), between August 2013 and April 2015. SR, SST, SSS showed to have significant negative relationships with latitude, while Chl showed positive relationship (p < 0.001; Supplementary Fig. S1). In particular, SSS displayed two sites, Chioggia and Goro, detached from the other sites and Chl presented the same detached trend in the sites of Goro and Cesenatico, diverging both SSS and Chl from a linear relationship with latitude (Supplementary Fig. S1a). The sites of Chioggia, Goro and Cesenatico are the sites located near the Po delta (Fig. 1), in which the high variability in SSS and Chl values is due to the freshwater inflows during seasons (Supplementary Fig. S1a). All the environmental parameters were significantly correlated with each other, in particular SST with SR and SSS (p < 0.001; Supplementary Fig. S1b).

**Table 1:** Environmental parameters.

Code	Latitude (°)	n	SR (W/m^2^) mean	CI	SST (°C) mean	CI	SSS (PSU) mean	CI	n	Chl (mg/m^3^) mean	CI
MO	45.7	1447	159.44	155.45–164.43	16.96	16.58–17.34	35.43	35.37–35.49	48	4.50	4.11–4.89
CH	45.2	1447	160.76	155.82–165.70	16.47	16.09–16.85	30.89	30.72–31.06	48	2.88	2.45–3.31
GO	44.8	1447	163.78	158.74–168.82	16.54	16.17–16.91	28.52	28.36–28.68	48	4.98	4.21–5.75
CE	44.2	1447	165.17	160.18–170.16	17.05	16.65–17.45	34.19	34.11–34.27	48	6.23	5.03–7.43
SB	43.1	1447	172.39	167.39–177.39	17.90	17.52–18.28	36.29	36.24–36.34	48	2.09	1.54–2.64
CA	41.9	1447	180.44	175.36–185.52	18.60	18.27–18.93	37.43	37.40–37.46	48	1.21	0.85–1.57

Mean annual values for solar radiation (SR), sea surface temperature (SST), sea surface salinity (SSS) and Chlorophyll concentration (Chl) from 2011 to 2014. n = number of collected data, daily data for SR, SST and SSS and monthly data for Chl; CI = 95% confidence interval. Values for each site, in order of decreasing latitude: MO (Monfalcone), CH (Chioggia), GO (Goro), CE (Cesenatico), SB (San Benedetto), CA (Capoiale).

Shell lengths were homogeneous among sites (Kruskal-Wallis test, df = 5 and p > 0.05; Supplementary Table S1). Also age did not differ among sites both for external rings and internal bands (Kruskal-Wallis test, df = 5 and p > 0.05; Supplementary Table S1).

**Figure 3:**
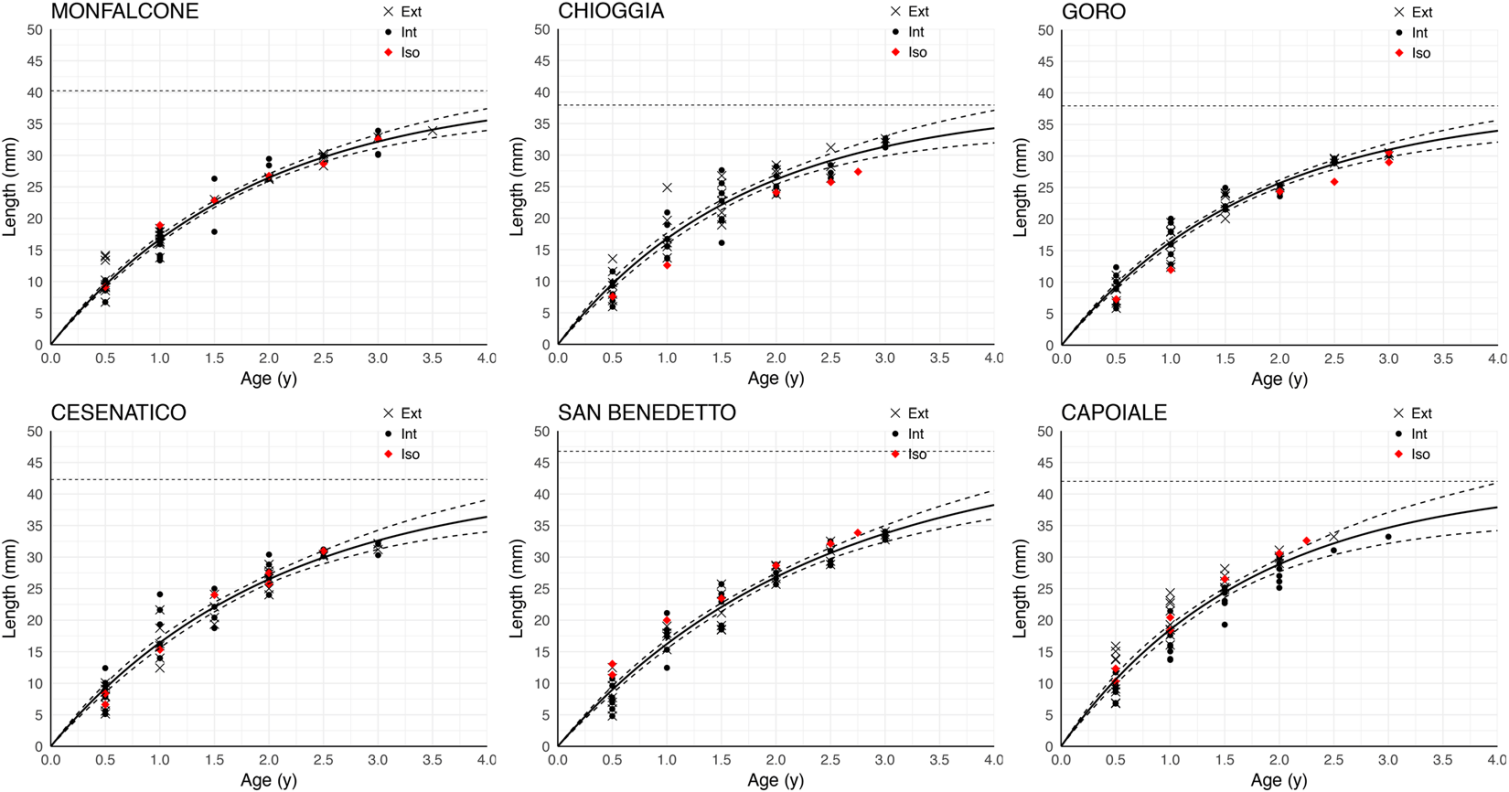
Von Bertalanffy growth curves. The generalised age-length von Bertalanffy growth curve in each site obtained by all data from the two ageing methods (counting of external rings representing with crosses and counting of internal rings representing with black dots). Red diamonds are figured as validation to the counting ageing methods fitting the curves. Dotted lines indicate the maximum expected shell length (L_inf_), dashed lines indicate the confidence intervals constructed through bootstrap method. Red points are the ages obtained from the δ^18^O profiles along the shell growth axis.

There were no significant differences among the growth curves obtained from the external and internal rings within each site (Supplementary Table S2), therefore, a generalised VBG curve was obtained for each site (Fig. 3). The δ^18^O values along the shell growth direction exhibited a roughly sinusoidal sequence of lower (summer) and higher (winter) values, and the number of observed seasons allowed estimation of age (Fig. 2c). Age from δ^18^O values validated the data from the other two ageing methods fitting the VBG curves (Fig. 3).

**Figure 4:**
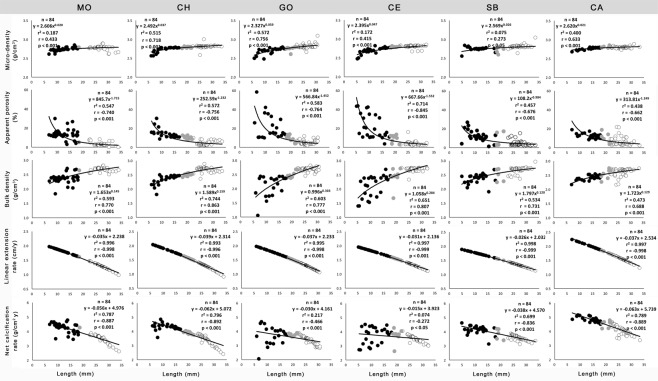
Relationships between shell skeletal and growth parameters and length. Black dots are immature clams (length <18 mm), grey dots are mature clams (>18 mm) and white dots are the clams of commercial size (>22 mm). n = number of individuals. r = Pearson’s determination coefficient. Sites are arranged in order of decreasing latitude: MO (Monfalcone), CH (Chioggia), GO (Goro), CE (Cesenatico), SB (San Benedetto), CA (Capoiale).

At each site, shell skeletal and growth parameters were significantly correlated with shell length (Fig. 4). At all sites bigger shells exhibited higher micro-density and bulk density and lower apparent porosity compared to smaller ones (Fig. 4). Moreover, linear extension rates and net calcification rates decreased with shell length at all sites (Fig. 4).

**Figure 5:**
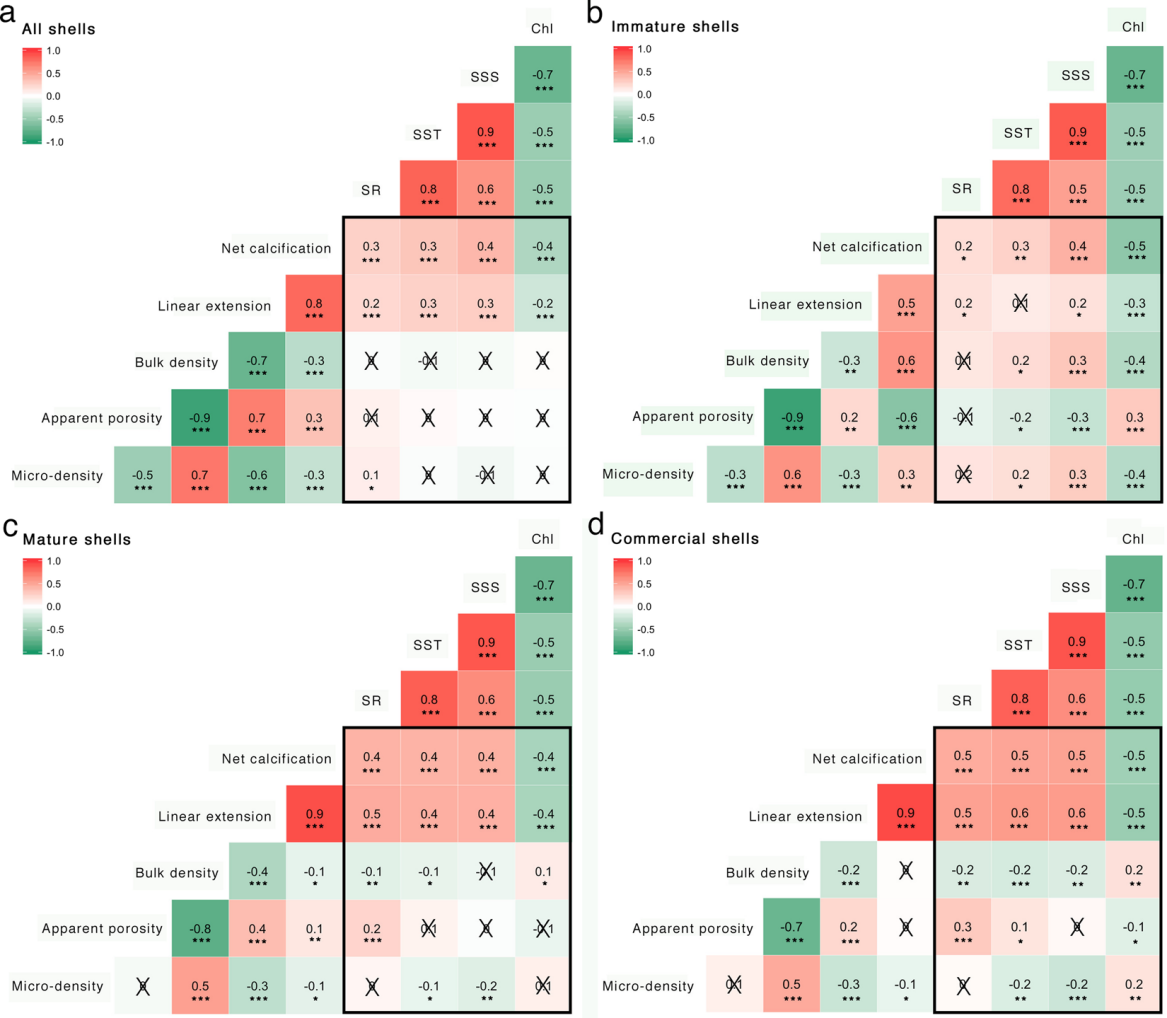
Correlation analysis between environmental and shell skeletal and growth parameters. (**a**) correlations in all shells. (**b**) Correlation in immature shells (<18 mm). (**c**) Correlations in mature shells (>18 mm). (**d**) Correlations in shells of commercial size (>22 mm). Scale colour bar and the corresponding written value in the plot indicate Spearman’s rho coefficient. *p < 0.05, **p < 0.01, ***p < 0.001. SR, solar radiation; SST, sea surface temperature; SSS, sea surface salinity; Chl, Chlorophyll concentration.

Variation of shell skeletal and growth parameters was then analysed in relation to environmental variables along the latitudinal gradient. Correlations were performed in the whole dataset of 84 shells for each site (Table 2, Fig. 5a). Apparent porosity and bulk density were not correlated with environmental parameters, while linear extension rate and net calcification showed significant positive correlations with SR, SST, SSS and negative correlations with Chl (Fig. 5a). Correlations with the environment were also performed in the immature and mature shells separately and in a subgroup of the mature shells including only shells of commercial size (>22 mm). In all groups, linear extension and net calcification were positively correlated with SR, SST, SSS and were negatively correlated with Chl, except extension rate which did not correlate with SST in immature shells (Fig. 5b, c, d; Supplementary Table S3 and Figs S2–S4). Micro-density, apparent porosity and bulk density showed no trends with SR in immature shells (Fig. 5b; Supplementary Table S3 and Fig. S2). In mature shells, apparent porosity showed no correlations with SST, SSS and Chl while bulk density correlated with SR, SST and Chl (Fig. 5c; Supplementary Table S3 and Fig. S3). In shells of commercial size apparent porosity positively correlated with SR, SST and negatively with Chl and bulk density correlated positively with Chl and negatively with SR, SST and SSS (Fig. 5d; Supplementary Table S3 and Fig. S4). Overall, comparing relationships between environmental and growth and skeletal parameters in shells of different size, environmental variables seemed to have a greater influence on shells of commercial size over 22 mm, in which we found 18 out 20 significant relationships (Fig. 5; Supplementary Table S3).

**Table 2:** Shell skeletal and growth parameters.

Site	n	Length (mm)	CI	Micro-density (g/cm^3^)	CI	Apparent porosity (%)	CI	Bulk density (g/cm^3^)	CI	Linear extension rate (cm/y)	CI	Net calcification (g/cm^2^ y)	CI
MO	84	21.26	19.59–22.94	2.76	2.75–2.77	7.60	6.22–8.98	2.55	2.51–2.60	1.50	1.44–1.55	3.78	3.68–3.89
CH	84	21.90	20.33–23.48	2.79	2.78–2.80	7.85	6.64–9.05	2.57	2.53–2.61	1.46	1.40–1.52	3.72	3.61–3.83
GO	84	22.05	20.63–23.47	2.78	2.76 -2.80	9.60	7.46–11.74	2.52	2.45–2.59	1.41	1.36–1.47	3.51	3.42–3.60
CE	84	21.42	19.76–23.07	2.76	2.73–2.79	10.05	7.70–12.39	2.49	2.41–2.57	1.48	1.43–1.53	3.61	3.52–3.70
SB	84	21.59	19.86–23.31	2.78	2.75–2.80	7.39	6.19–8.60	2.57	2.53–2.62	1.47	1.43–1.52	3.76	3.68–3.84
CA	84	22.59	21.29–23.90	2.79	2.78–2.80	8.12	7.05–9.20	2.57	2.53–2.60	1.69	1.64–1.74	4.31	4.22–4.40
K-W		NS		***		**		***		***		***	

n = number of samples; CI = 95% confidence interval. Sites are arranged in order of decreasing latitude: MO (Monfalcone), CH (Chioggia), GO (Goro), CE (Cesenatico), SB (San Benedetto), CA (Capoiale). K-W = Kruskal-Wallis rank test, NS = not significant, ** p < 0.01, *** p < 0.001.

## Discussion

In this study, we successfully used shell external and internal growth rings to estimate the age of *C*. *gallina* and build growth curves for this species at six sites along a wide latitudinal gradient. In some samples, the annual growth rings were hardly to determine because the shell external surface was smooth or damaged and because the blue-staining pattern in the internal section was not very defined, highlighting ambiguous internal growth lines. Both methods were validated by δ^18^O profiles along shell growth direction, suggesting the methods were appropriate and fairly accurate for the age estimation of *C*. *gallina* specimens. This work was the first attempt to determine the age from the internal shell section of *C*. *gallina* using the Mutvei’s solution. We can conclude that counting of internal growth rings, after shell sectioning and Mutvei’s treatment is a time-consuming method with no evident growth pattern at all times, suggesting to better adopt external growth rings and oxygen isotope analyses in future age studies of *C*. *gallina*. Our values are in conformity with the estimated maximum shell length and growth constant of *C*. *gallina* from previous studies in the Adriatic Sea^35^, in the Western Mediterranean Sea^19^ and in the Algarve coast^17^. The differences reported in maximum asymptotic length (L_inf_) and von Bertalanffy growth constants (*K*) among sites (Supplementary Table S2) are probably due to local environmental conditions, as already suggested by previous studies on the growth of *C*. *gallina* conducted in different areas^17^. In previous studies, the eastern populations of this species, from the Marmara and Adriatic Sea, showed greater longevities than western populations along the Spanish coast and the Algarve coast^17,32^.

As previously observed for molluscs and for other organisms^36,37^, *C*. *gallina* extension rate decreased with increasing length. The reduction in net calcification rates with size was determined by decreasing linear extension rates, partly countered by increasing bulk density. A higher apparent porosity was observed in shells of small size and it sharply decreased to less than 20% approaching the length at sexual maturity (about 18 mm)^38^. High porosity influenced bulk density which was conversely lower in small size shells. This suggests that during the first year of life, *C*. *gallina* seems to promote porosity, enabling it to keep higher linear extension rates in order to reach the size at sexual maturity. Although juveniles are more vulnerable than adults to most predators, a denser skeleton could limit the rate of body growth, increasing the time spent at smaller, non reproductive sizes, while more porous ones could lead to an increase in shell’s linear extension rate allowing *C*. *gallina* to reach size at sexual maturity faster^39^. *C*. *gallina* is a gonochoric species with external spawning thus having a larger shell could mean more space available for gonads. From about 20 mm in length, *C*. *gallina* seems to change its biomineralization behavior, showing small variations in apparent porosity and bulk density and a continuous decrease in linear extension rate and net calcification. Moreover, bigger and older individuals fully allocated net calcification in making denser shells likely to be less vulnerable to predators, by depressing linear extension rate^39^.

A previous study conducted along the same latitudinal gradient in the Adriatic Sea showed that solar radiation and sea surface temperature directly affected shell skeletal properties in specimens of *C*. *gallina* of commercial size, showing more porous and less dense shells in the most irradiated and warm populations^26^, but these trends were not analysed in specimens less than 25 mm long. This is the first study investigating shell skeletal and growth parameters during the lifespan of the clam *C*. *gallina* and in relation to solar radiation, temperature, salinity and chlorophyll concentration in both immature and mature shells. Environmental parameters seemed to have a greater influence on large shells over 22 mm long. Large clams with lower growth rates tend to have higher standard metabolic rates (SMR) as confirmed by the “principle of allocation” theory^40^, such as the potential tradeoff in the allocation of energy between growth and maintenance metabolism that result to be negatively correlated. Earlier studies in marine bivalves showed that individuals with higher SMR are less resistant to environmental stress and this is in agreement with our findings that mature shells were more influenced by environmental variables than immature shells^40,41^. Individuals with higher SMR, like mature clams, must depend more on their reserves to sustain vital functions and support physiological responses to stress; instead individuals with lower energetic requirements, like immature clams, have a surplus of energy with which to withstand stressful conditions^41^.

Net calcification rates increased towards southern populations, with increasing SR, SST and SSS and lower Chl, in immature and mature shells. The immature shells allocated increased calcification rates on bulk density. A possible explanation could be that denser clams could make them less vulnerable to predation, which often affects early life stages^42^. This hypothesis is in agree with the general trend of reducing vulnerability with incrementing prey size reported for example for molluscs preyed by decapods^43^. Predation likely relies on the ability of the predators to crack or perforate the shells and changes in shell characteristics to enhance strength, such as increase in bulk density, could lead to higher survival of small individuals, particularly where predation pressure is high^42^. Very little is known on predatory species of *C*. *gallina* in the Adriatic Sea; some of these are the starfish, *Astropecten spp*., the gastropod *Neverita josephinia* and the fish, *Gobius niger* and *Lithognathus mormyrus*^44^. However, further studies on the population density of these species along the latitudinal gradient in the Adriatic Sea are needed to confirm this hypothesis. The differences in shell density that we observed along the gradient could depend on different mineralization rates driven by environmental parameters, especially by temperature. Towards South, immature clams could raise their shell density due to a decrease in the energetic costs of shell formation with increasing aragonite saturation state in warmer waters^45^. In contrast to immature clams, in mature ones, the increase in calcification rates towards Southern populations was invested on increasing linear extension rates. A possible explanation could be that bigger shells could make them more fertile, by suppling more space for gonads, but the hypothesized biological significance for this calcification pattern has still to be investigated.

Since *C*. *gallina* is an infaunal bivalve, the effect of SR along the latitudinal gradient is probably related to other abiotic and/or biotic parameters, such as temperature and phytoplankton density. The increase in net calcification rates with increasing SST could be due to a decrease in the energetic costs of shell formation with increasing aragonite saturation state in warmer waters^45^. Studies on molluscs highlight that calcification increases with aragonite saturation state^46^. In this study, SST does not exceed 19 °C of mean annual sea surface temperature in the southernmost site and producing carbonate shells could be less expensive than in colder sites with mean SST of 16 °C. There are still no aragonite saturation data available along the latitudinal gradient to deeply understand relationships between shell calcification and seawater chemistry in the Adriatic Sea.

Chlorophyll *a* concentration is a good food proxy for clams, based on the assumption that phytoplankton is the main component of suspension feeding bivalves diet^47^. The Adriatic Sea is characterized by a lower phytoplankton concentration in the South compared to the North^48^. Previous results indicate that the clam *Venus verrucosa* grew faster in areas with high Chlorophyll *a* concentrations in the Eastern Adriatic Sea^49^ and that food availability played an important role in determining the growth rates of population of bivalves^50^. Despite growth increases as a function of food concentration^51^, in this study shell linear extension and net calcification rates resulted to be lower with high Chl in all size shells, suggesting there could be other environmental parameters that synergistically affected the growth of *C*. *gallina*. Indeed, along the latitudinal gradient in the Adriatic Sea the enhanced growth of *C*. *gallina* could be due to higher temperature and salinity that mitigate the contingent lower food availability.

Here we show that in the northern sites under Po delta influence, in particular in the site of Goro, linear extension and calcification rates of *C*. *gallina* were reduced compared to Southern sites. One possible explanation could be non-optimal salinity conditions (SSS < 30). The Po river flow reduces salinity, exposing *C*. *gallina* to strong seasonal variations in salinity values with intense reductions in autumn and rises in summer^52^, especially in the site of Goro, while the other sites show increasing salinity moving from Goro towards North and South. While *C*. *gallina* is an euryhaline species with a high ability to acclimatize to extremely brackish conditions^53^, suboptimal salinity may constitute a stressor leading to modify biochemical mechanisms, such as incrementing their antioxidant defences to face the higher oxidative stress^54^ and to vary physiological responses, such as valve closure, reducing feeding activity and slower growth rates^54,55^. Moreover, lower salinity water also leads to lower aragonite saturation state^56^, with increasing cost of calcification^57^. Another possible explanation could depend on eutrophication. In the Adriatic Sea, the marine environment is strongly affecting by the input of Po river nutrients, that influence sea water transparency and lead to anoxic events^58^. Slow growth rates associated with eutrophicated habitats have been previously recorded for the bivalve *Cerastoderma edule*^59^ and for the bivalve *Austrovenus stutchburyi*^60^ and these studies seem to confirm the results found for *C*. *gallina* that showed higher extension and calcification rates in oligotrophic conditions. Moreover, silt and clay that characterise the bottom of the Po delta area could interfere with the feeding mechanism, leading to low extension and net calcification rates and growth rates were found to be higher in sand than mud^60^. Thus, the observed reduction in extension and calcification rates of *C*. *gallina* in those sites that are most influenced by the Po delta could depend on a number of factors acting synergistically. However, further studies are needed to test these hypotheses.

The geographic information of growth reported in this paper, with an increase in calcification and linear extension rates towards Southern sites, could suppose that fisheries in the Southern sites could be characterised by bigger clams with a significant raising of inside edible mass compared to the clams of the Northern sites. But further analysis on the clam flesh is essential to investigate this hypothesis.

A complex pattern of interactions between the organism and several habitat conditions (e.g. temperature, salinity, food availability) shapes the growth of the species over time and space. Investigating these relationships and modelling the environmental control of marine species growth is essential in sustainable management of coastal ecosystems. Valuable insights for developing ecosystem-based management tools of aquaculture activities derives from studies providing relevant information on environmental conditions affecting mollusc growth^61^. In addition, knowledge of the growth rates allows a proper management of bivalve fisheries and the observations highlight in this study can be used in predictive models to explore the evolution of *C*. *gallina* resource exploited during time under extended changes in their habitat conditions, for example seawater temperature, salinity or food. The present paper, together with continue monitoring of clam stocks, is aimed at providing in-depth insights on age and growth, considered the prerequisite to generate the information on recruitment, longevity, mortality, and their application in developing sustainable and efficient fisheries management policies imposed by the Ministry of Agricultural, Food and Forestry Policies in Italy; (e.g. DM 27/12/2016 on clams discard in the Italian stocks).

## Conclusions

Differences found in shell skeletal parameters, especially apparent porosity and bulk density, with length could be due to different biomineralization patterns between immature and mature shells of *C*. *gallina*. Before reaching sexual maturity, *C*. *gallina* seemed to promote porosity enabling it to keep higher linear extension rates in order to reach the size at sexual maturity faster, while after sexual maturity shells seemed to depress linear extension rate and make denser shells.

Moving far from the Po delta towards South, warmer seawater, low fluctuations in salinity and oligotrophic conditions suggested that these environmental conditions may be most favourable for the clam *C*. *gallina*, leading to higher net calcification rates. Net calcification rates were significantly reduced in sites around the Po delta, possibly as a result of lower temperature and reduced salinity that increase the energetic costs of shell formation with decreasing aragonite saturation state. Net calcification could also be reduced as a result of increased eutrophication and silt and clay of the bottom driven by the river discharges that could interfere with the feeding mechanisms. The present study therefore points out the importance of considering multiple environmental parameters to investigate bivalve growth. In addition, knowledge of the growth rates allows a proper management of bivalve fisheries. Given the great socio-economic relevance of *C*. *gallina* in all the Italian Adriatic coasts, studies like this one are crucial to guarantee a knowledge-based management of this important resource.

## Materials and Methods

Between August 2013 and April 2015, specimens of *C*. *gallina* were collected from six sites along a latitudinal gradient in the Adriatic Sea from 45°42′N to 41°55′N (Fig. 1). Clams were sampled at each site using hydraulic dredges at 3–5 m depth. 84 shells of different size from each site were used for the analyses. Shells were divided in three groups: immature (up to 18 mm), mature shells^62^ (over 18 mm) and commercial shells (over 22 mm, new experimental commercial size adopted from January 2017).

Skeletal apparent porosity (percentage of the pore volume connected to the external surface; %) and micro-density (mass per unit volume of the material which composes the shell, excluding the volume of pores; g cm^−3^) were measured by buoyant weight analysis, using a density determination kit Ohaus Explorer Pro balance (±0.1 mg; Ohaus Corp., Pine Brook, NJ, USA)^26^. Measurements required for calculating apparent porosity and micro-density were^26^:

ρ density of the fluid (double distilled water: 0.998 g cm^−3^ at 20 °C and 1 atm)

DW dry mass of the shell

SW saturated mass of the shell = mass of the shell plus mass of the water enclosed in its pores

BW buoyant mass of the shell = mass of the shell fully saturated with water minus mass of the water displaced by it.

$${V}_{{MATRIX}}=\frac{DW-BW}{\rho }{\rm{matrix}}\,{\rm{volume}}={\rm{volume}}\,{\rm{of}}\,{\rm{the}}\,{\rm{shell}},\,{\rm{excluding}}\,{\rm{the}}\,{\rm{volume}}\,{\rm{of}}\,{\rm{its}}\,{\rm{pores}}$$

$${V}_{{PORES}}=\frac{SW-DW}{\rho }{\rm{pore}}\,{\rm{volume}}={\rm{volume}}\,{\rm{of}}\,{\rm{the}}\,{\rm{pores}}\,{\rm{in}}\,{\rm{the}}\,{\rm{shell}}$$

$${V}_{{TOT}}={V}_{{MATRIX}}+{V}_{{PORES}}\,{\rm{volume}}={\rm{volume}}\,{\rm{of}}\,{\rm{the}}\,{\rm{shell}}\,{\rm{including}}\,{\rm{its}}\,{\rm{pores}}$$

Additionally, the following skeletal parameters were calculated^26^:

$$\mathrm{Micro} \mbox{-} \mathrm{density}({\rm{matrix}}\,{\rm{density}})={\rm{DW}}/{{\rm{V}}}_{{\rm{MATRIX}}}$$

$${\rm{Bulk}}\,{\rm{density}}=\,{\rm{DW}}/{{\rm{V}}}_{{\rm{TOT}}}$$

$${\rm{Apparent}}\,{\rm{porosity}}=({{\rm{V}}}_{{\rm{PORES}}}/{{\rm{V}}}_{{\rm{TOT}}})\times 100$$

In addition, clam shell length (anterior-posterior maximum distance) was obtained with ImageJ software after data capture of each shell shape with a scanner and dry shell weight was measured using an analytical balance (±0.1 mg)^26^.

Bulk density (shell mass/volume ratio, including the volume of pores) was also measured by buoyant weight analysis. Shell linear extension rates were obtained with the length/age ratio (cm y^−1^), while the net calcification rate (mass of CaCO_3_ deposited per year per unit area g y^−1^ cm^2^) was calculated for each shell by the formula: net calcification (g cm^−2^ y^−1^) = bulk density (g cm^−3^) x shell extension (cm y^−1^)^63^.

Age was measured in a subsample of 30 shells of different size in each site, by using three methods: shell surface growth rings (Fig. 2a), shell internal bands (shell cross-sections and Mutvei’s solution; Fig. 2b) and stable δ^18^O composition (Fig. 2c; see ESM for the ageing methods details). By counting the total number of visible external and internal rings in each shell, the age-length keys were obtained for the two methods and fitted with the von Bertalanffy growth (VBG) functions, using a non-linear model that provides estimates of the parameters in the VBG equation through bootstrap method:

$${L}_{t}={L}_{inf}[1-{e}^{-k(t)}]$$

where L_t_ is individual length at age t, L_inf_ is asymptotic length (maximum expected length in the population), K is a growth constant, t is the age of the individual. Two growth curves for each sites were produced and a chi-square test of maximum likelihood ratios was used to examine the significance of differences in growth functions between the two ageing method. Kimura’s method allows the testing of several hypotheses to compare the two curves by analysing one or more growth parameters simultaneously. For these purposes, the FSA (Simple Fisheries Stock Assessment Methods) and the Fishmethods packages in R studio were used.

If no differences were revealed between VBG curves from shell surface growth rings and internal bands, generalised growth curves for each site were constructed by merging age-length keys from both methods and the resulted generalised VBG function for each site were taken into account for extrapolating age in all the 84 shells. Age were calculated from the inverse of generalised VBG function of each site:

$$t=\frac{1}{k\ast \,\mathrm{ln}(\frac{{L}_{inf}}{{L}_{inf}-L})}$$

To validate the data from the two counting rings methods and the generalised VBG curves, oxygen isotopic measurements (δ^18^O) were carried out at the Godwin Laboratory for Palaeoclimate Research, Department of Earth Sciences, University of Cambridge. δ^18^O measurements were carried out on “spot” samples collected from the prismatic layer and from the cross-lamellar layer and drilled in sequence along the shell growth direction by means of a 0.5 mm dental diamond drill^15^. Dried homogenized powdered samples were treated with helium then added an acidified solution consisting of 104% orthophosphoric acid and left to react for 1 hour at 70 °C. Each sample was then analysed using a Thermo Gasbench preparation system attached to a Thermo Delta V Advantage mass spectrometer in continuous flow mode. Age resulted from counting lighter δ^18^O (summer) and heavier δ^18^O (winter) peaks were then plotted with the age-length key from the two ageing methods.

### Environmental parameters

Solar radiation (SR; W m^−2^), Sea Surface Temperature (SST; °C) and Sea Surface Salinity (SSS; PSU) data were obtained for each site from the Euro-Mediterranean Center on Climate Change data banks (CMCC http://oceanlab.cmcc.it/afs/)^26^. Mean annual SR, SST and SSS were calculated from daily values measured from July 2011 to June 2015 (number of daily values = 1447 for each site, instead of 1460 days for 4 years for 13 days missing data), to cover the almost full *C*. *gallina* lifespan of two-three years for the samples under investigation. For the same period, a mean annual Chlorophyll concentration (Chl; mg/m^3^) was calculated from monthly values of Chl, obtained for each site (48) from the *GlobColour data* (http://globcolour.info) by ACRI-ST, France (http://hermes.acri.fr).

## Supplementary information

Supplementary Information

## Data Availability

The dataset generated and analysed during the current study is available from the corresponding author on reasonable request.
