# Supplementary material for: Environmental influence on calcification of the bivalve Chamelea gallina along a latitudinal gradient in the Adriatic Sea
Source: Sci Rep. 2019 Aug 1;9:11198. doi: 10.1038/s41598-019-47538-1 (PMC6671978; doi:10.1038/s41598-019-47538-1)

**Supplementary Information from**

**Environmental influence on calcification of the bivalve *Chamelea gallina* along a latitudinal gradient in the Adriatic Sea**

**Arianna Mancuso^1,2^, Marco Stagioni^2^, Fiorella Prada^1^, Daniele Scarponi^3^, Corrado Piccinetti^2^, Stefano Goffredo^1,2 *^**

***corresponding author: E-mail** [**s.goffredo@unibo.it**](mailto:s.goffredo@unibo.it)

1Marine Science Group, Department of Biological, Geological and Environmental Sciences, University of Bologna, Via Selmi 3, I-40126 Bologna, Italy, European Union

2Laboratory of Fisheries and Marine Biology at Fano, Department of Biological, Geological and Environmental Sciences, University of Bologna, Viale Adriatico 1/N, I-61032, Fano (PU), Italy, European Union

3Department of Biological, Geological and Environmental Sciences, University of Bologna, Via Selmi 3, I-40126 Bologna, Italy, European Union

**METHODS**

Shell ageing methods details

For counting surface external rings, shells were scanned in a transmitted light to enhance contrast of surface’s ridge and highlight bands at different density that can be considered growth external rings (Fig. 2a). In order to estimate age by means of shell sectioning and Mutvei’s solution, the valves were embedded in epoxy resin under vacuum at room temperature, followed by 24 h hardening. For counting internal bands, the shells were sectioned along the anterior-posterior axis, from the umbo to the ventral margin, using an electrodeposited diamond cutting blade (Fig. 2b). Sections were subsequently mounted on glass slides with Crystalbond 509 Clear, ground with SiC paper (600 and 2500 mesh) and polished with abrasive allumina compound (3M Perfect-it III Extrafine Paste). Finally, the sections were ultrasonically cleaned, rinsed in purified water and dried. The original Mutvei’s solution consists of 500 ml 1% acetic acid, 500 ml 25% glutaraldehyde and ca. 5 to 10 g alcian blue powder ^1^ while the solution used in this study was made of acetic acid, that removed the carbonate very gently and assisted formaldehyd in stabilizing the organic compounds, formaldehyd fixative that traps proteins and toluidine blue for staining. Shell sections were covered with some drops of Mutvei’s solution for 30 minutes at 37–40°C and shaken with a vortex every five minutes to facilitate bubbles escape. Immediately after Mutvei’s removal, the etched sections were carefully rinsed with purified water and allowed to air-dry. After Mutvei’s solution shell sections with shadings of blue revealed growth structures, with annual growth lines stood out as etch-resistant ridges, loosely blue-stained, while growth increments were etched and darker blue-stained. Shell sections were then examined under oblique light at low magnification and photographed with a digital camera (PowerShot G12; Canon, Tokyo, Japan), in order to identify internal growth bands (Fig. 2b). For each shell, the photos were merged together as a patchwork to show an overall view of the entire section (Fig. 2b).

To validate the data from the two counting rings methods, oxygen isotopic measurements (δ^18^O) were carried out on “spot” samples collected in sequence from the umbo to the ventral edge of the shells for each site (Fig. 2c). Dried homogenized powdered samples were treated with helium then added an acidified solution consisting of 104% orthophosphoric acid and left to react for 1 hour at 70 °C. Each sample was then analyzed using a Thermo Gasbench preparation system attached to a Thermo Delta V Advantage mass spectrometer in continuous flow mode. Each run of samples was accompanied by 10 reference carbonates (Carrara Z) and 2 control samples (Fletton Clay). Carrara Z has been calibrated to VPDB using the international standard NBS19. Age resulted from counting lighter δ^18^O (summer) and heavier δ^18^O (winter) peaks were then plotted with the age-length key from the two ageing methods (Fig. 3).

Statistical analyses

Levene’s test was used for testing homogeneity of variance and Kolmogorov-Smirnov’s test was used for testing normality of variance for both environmental and shell parameters. Since the assumptions for parametric statistics were not fulfilled, the non-parametric Kruskal-Wallis equality-of-populations rank test was used to test the significance of the differences among sites for environmental variables and shell parameters. Pearson’s correlation coefficient (r) was used to correlate shell skeletal and growth parameters with respect to shell length in each site, while Spearman’s rank correlation coefficient (rho) was used for the relationships between shell parameters and environmental parameters. All analyses were computed using R Studio Software (RStudio Team, 2016).

**References**

1. Dunca, E., Fiebig, J., Pfeiffer, M. & Schöne, B. R. Mutvei’s solution: An ideal agent for resolving microgrowth structures of biogenic carbonates. *Palaeogeogr. Palaeoclimatol. Palaeoecol.* **228,** 149–166 (2005).

**Supplemental Tables**

**Table S1. Length and age data.** Mean length and age determined from the external rings and internal growth bands counting in each site. n = number of samples; CI = 95% confidence interval. Sites are arranged in order of decreasing latitude: MO (Monfalcone), CH (Chioggia), GO (Goro), CE (Cesenatico), SB (San Benedetto), CA (Capoiale). K-W = Kruskal-Wallis equality-of-populations rank test, NS = not significant.


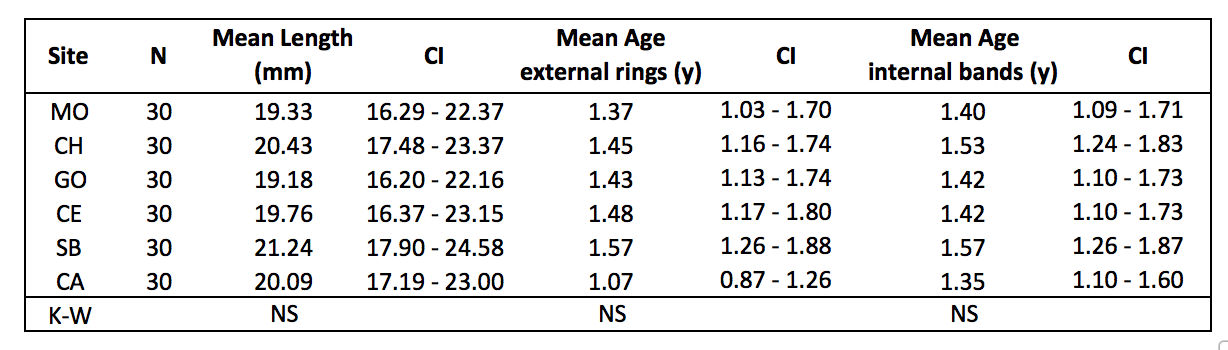


**Table S2. Von Bertalanffy growth parameters.** Linf and K estimated from the Von Bertalanffy growth function for external and internal bands and by pooling the data of the two methods (generalised). CI = 95% confidence interval. Sites are arranged in order of decreasing latitude: MO (Monfalcone), CH (Chioggia), GO (Goro), CE (Cesenatico), SB (San Benedetto), CA (Capoiale).


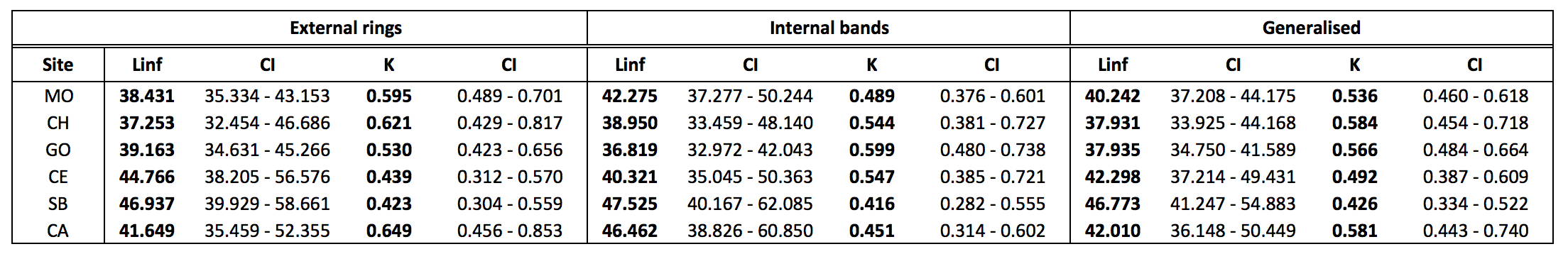


**Table S3. Linear regression and correlation analysis between environmental and shell skeletal and growth parameters.** rho, Spearman’s rho coefficient. rho and p-value are shown only when Kruskal-Wallis test is significant. Regression parameters are shown only where the linear relationship is significant. SR, solar radiation; SST, sea surface temperature; SSS, sea surface salinity; Chl, Chlorophyll concentration; SE, standard error.


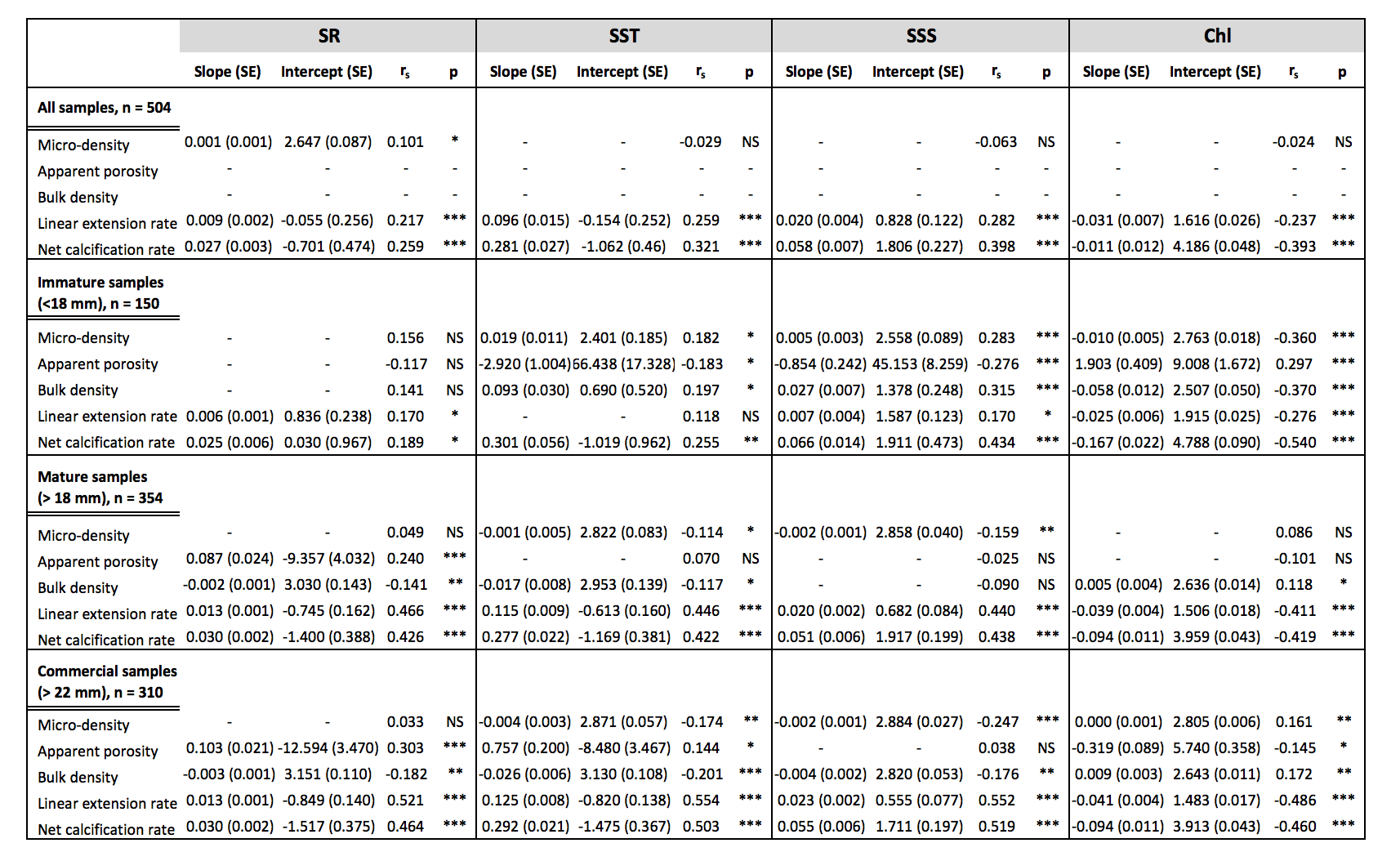


**Supplemental Figures**


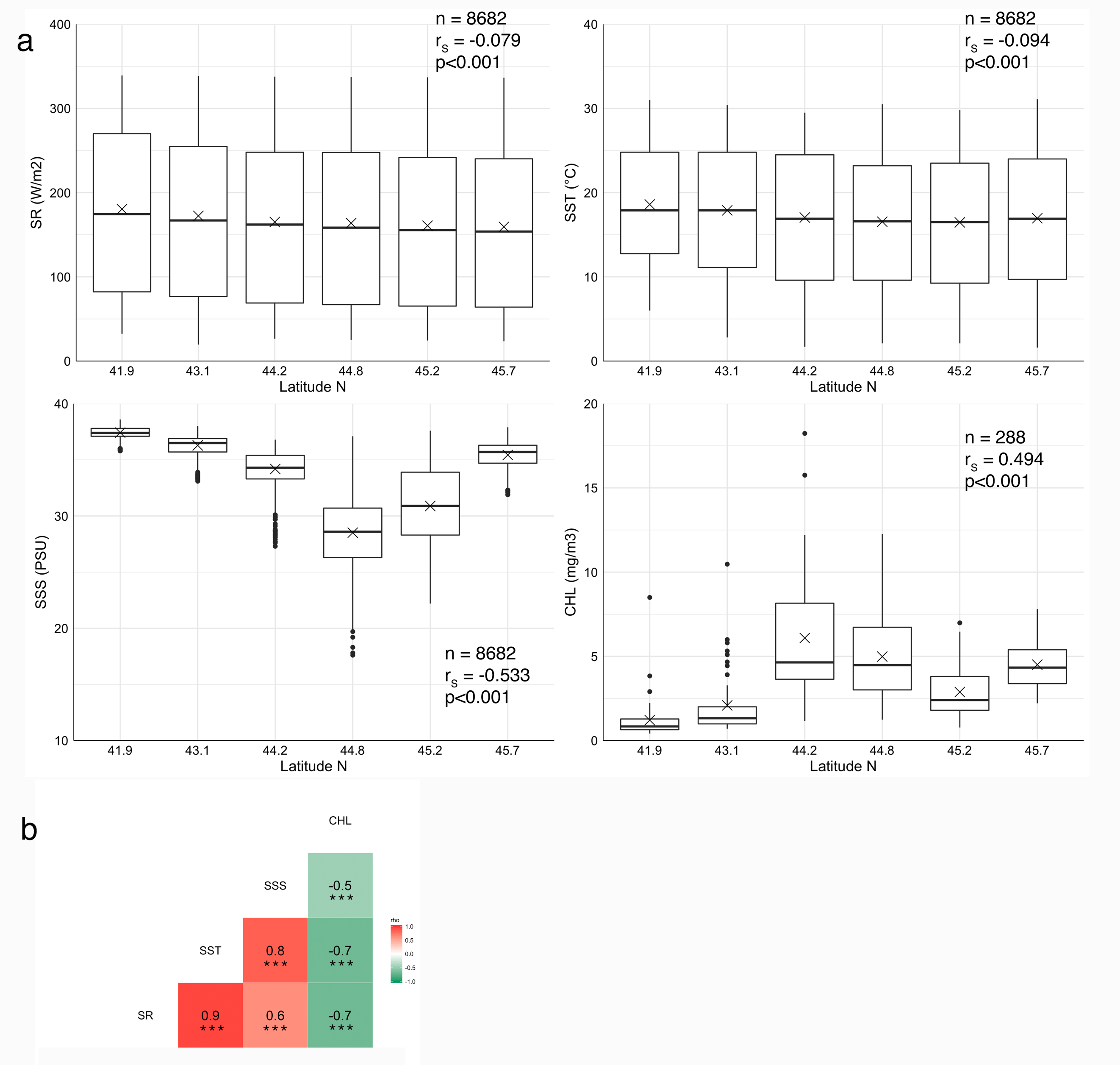


**Figure S1**. **Environmental parameters.** a. Relationship between environmental parameters and the latitude of study sites along the coast of Italy. The crosses indicate the mean annual values. n = daily values for SR, SST and SSS and monthly values for Chl; r_S_ = Spearman’s correlation coefficient. b. Correlations among environmental parameters. rho = Spearman’s correlation coefficient. All the correlations are significant, p<0.001*** (adjust p-values for multiple comparisons using correction of Holm, 1979). SR, solar radiation; SST, sea surface temperature; SSS, sea surface salinity; Chl, chlorophyll concentration.

Chl


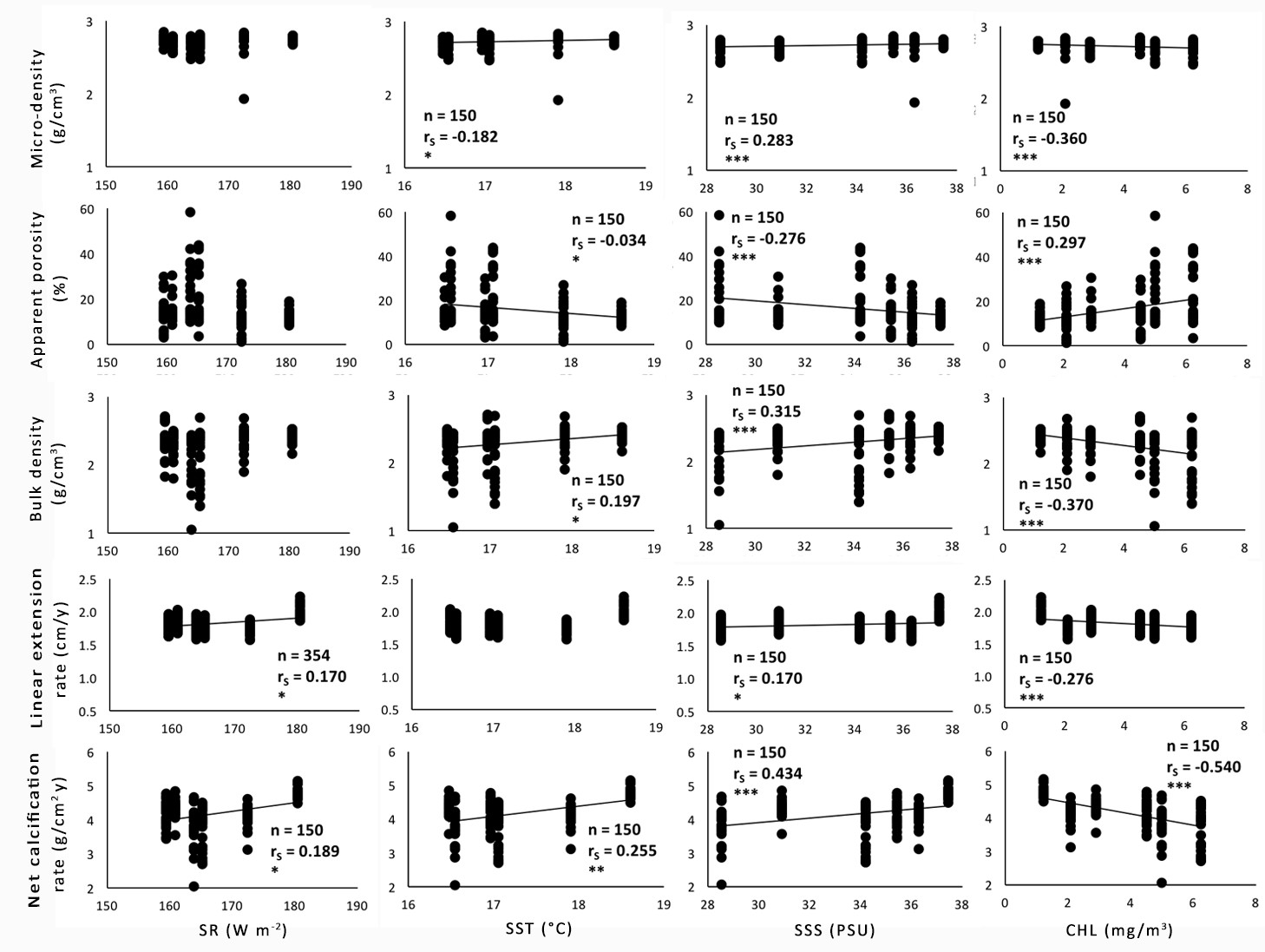


**Figure S2. Relationships between skeletal and growth parameters and environment in immature shells (< 18 mm).** r_S_ = Spearman’s determination coefficient. *p<0.05; **p<0.01; ***p<0.001. Linear regression and correlations are listed in Table S3. n = number of individuals. SR, solar radiation; SST, sea surface temperature; SSS, sea surface salinity; Chl, chlorophyll concentration.

**Figure S3. Relationships between skeletal and growth parameters and environment in mature shells (> 18 mm).** r_S_ = Spearman’s determination coefficient. *p<0.05; **p<0.01; ***p<0.001. Linear regression and correlations are listed in Table S3. n = number of individuals. SR, solar radiation; SST, sea surface temperature; SSS, sea surface salinity; Chl, chlorophyll concentration.


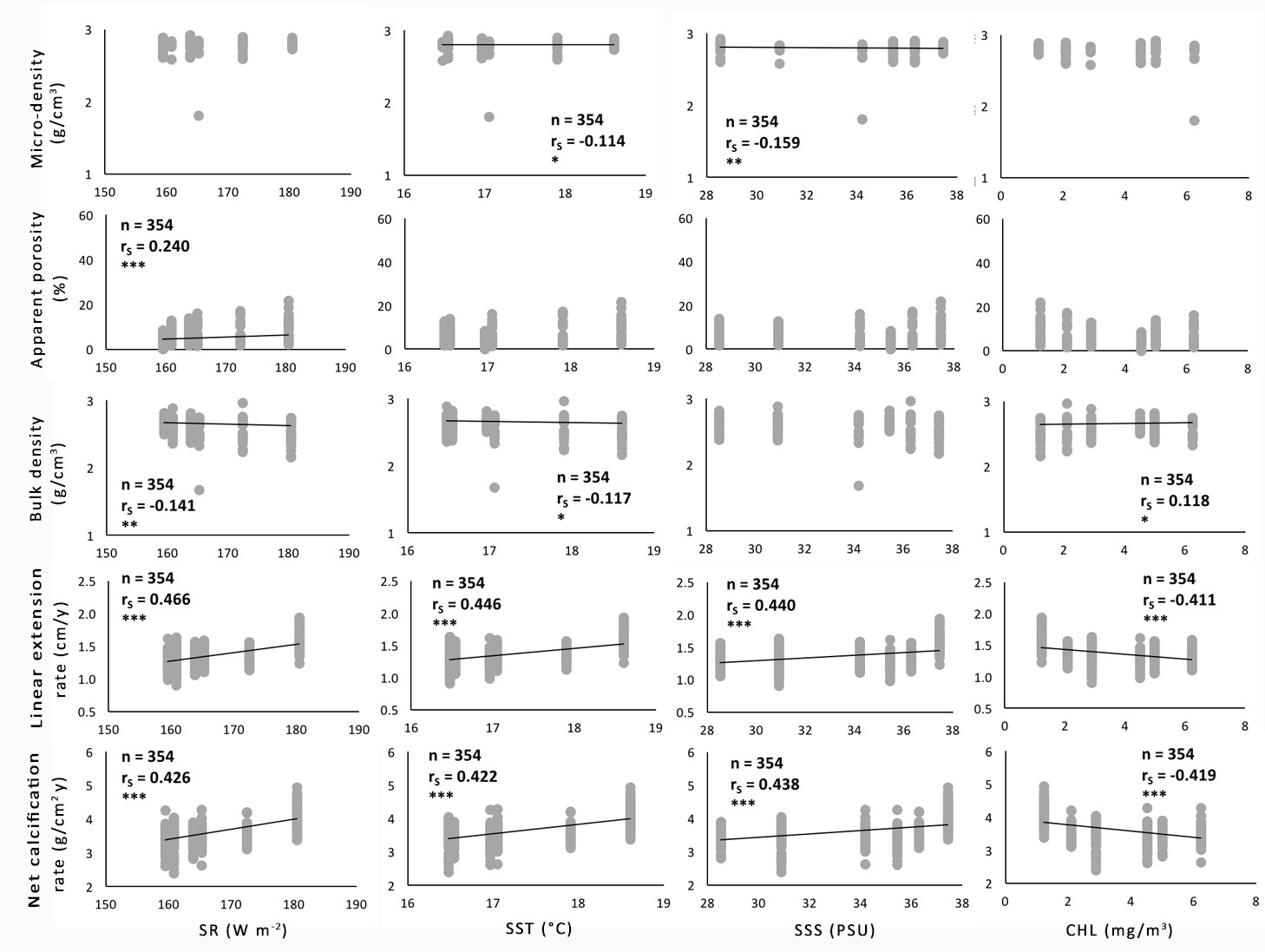


**Figure S4. Relationships between skeletal and growth parameters and environment in shells of commercial size (> 22 mm).** r_S_ = Spearman’s determination coefficient. *p<0.05; **p<0.01; ***p<0.001. Linear regression and correlations are listed in Table S3. n = number of individuals. SR, solar radiation; SST, sea surface temperature; SSS, sea surface salinity; Chl, chlorophyll concentration.


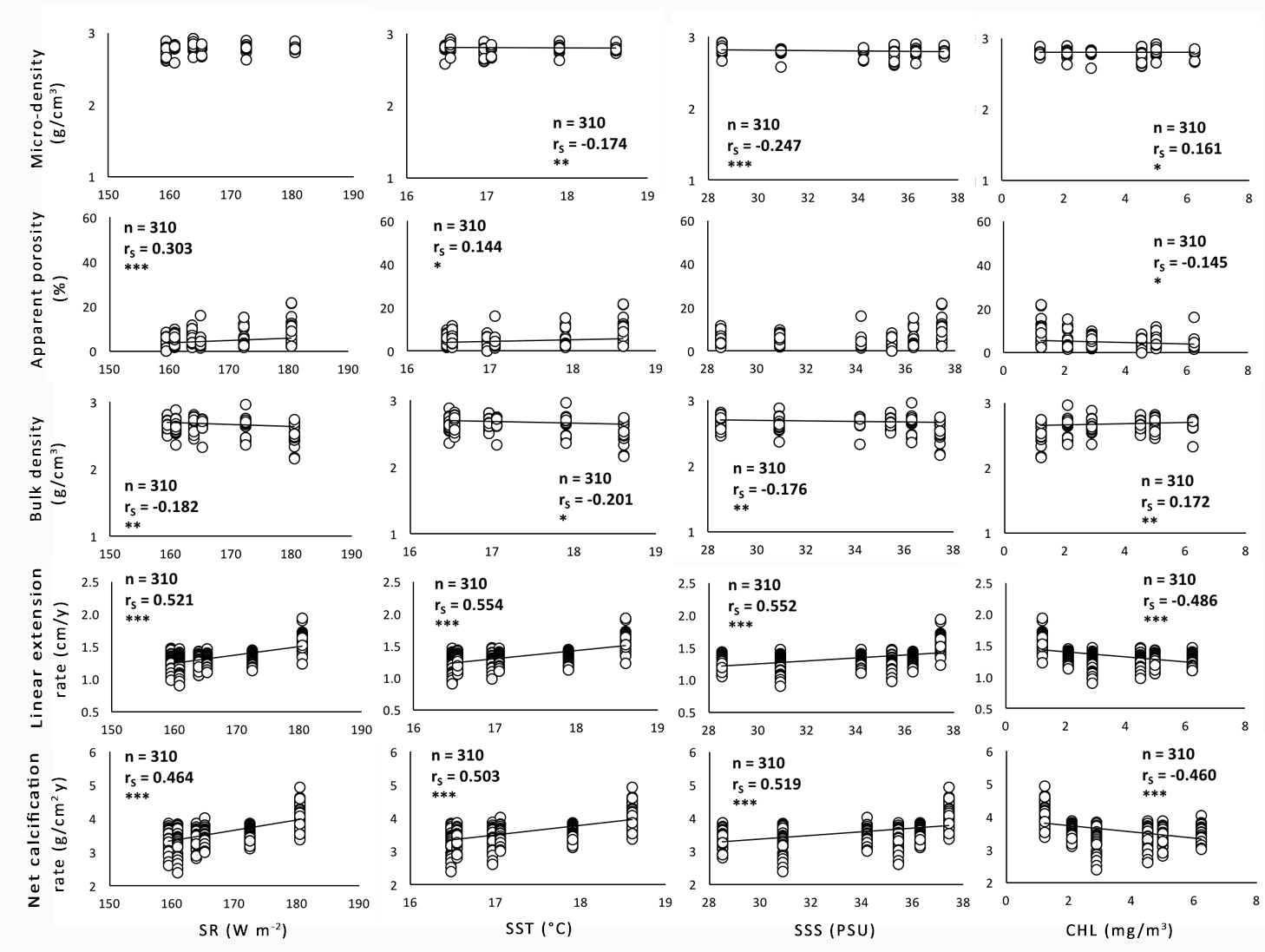

Supplement: Supplementary file 1 — Supplementary Information [file 41598_2019_47538_MOESM1_ESM.docx]
